# A Managerial Approach towards Modeling the Different Strains of the COVID-19 Virus Based on the Spatial GeoCity Model

**DOI:** 10.3390/v15122299

**Published:** 2023-11-23

**Authors:** Yaroslav Vyklyuk, Denys Nevinskyi, Valentyna Chopyak, Miroslav Škoda, Olga Golubovska, Kateryna Hazdiuk

**Affiliations:** 1 Department of Artificial Intelligence, Lviv Polytechnic National University, 79000 Lviv, Ukraine; 2 Department of Electronics and Information Technology, Lviv Polytechnic National University, 79000 Lviv, Ukraine; nevinskiy90@gmail.com; 3 Department of Clinical Immunology and Allergology, Danylo Halytsky Lviv National Medical University, 79010 Lviv, Ukraine; chopyakv@gmail.com; 4 Department of Management and Accounting, DTI University, 533/20, 018 41 Dubnica nad Váhom, Slovakia; skoda@dti.sk; 5 Department of Infectious Diseases, National Medical University by A. A. Bogomolets, 02000 Kyiv, Ukraine; ogolubovska@gmail.com; 6 Department of Computer Systems Software, Yuriy Fedkovych Chernivtsi National University, 58012 Chernivtsi, Ukraine; kateryna.gazdyik@gmail.com

**Keywords:** multi-agent system, GEO-spatial simulation model, COVID-19, modelling, geo-object, real-time simulation

## Abstract

This study proposes a modification of the GeoCity model previously developed by the authors, detailing the age structure of the population, personal schedule on weekdays and working days, and individual health characteristics of the agents. This made it possible to build a more realistic model of the functioning of the city and its residents. The developed model made it possible to simulate the spread of three types of strain of the COVID-19 virus, and to analyze the adequacy of this model in the case of unhindered spread of the virus among city residents. Calculations based on the proposed model show that SARS-CoV 2 spreads mainly from contacts in workplaces and transport, and schoolchildren and preschool children are the recipients, not the initiators of the epidemic. The simulations showed that fluctuations in the dynamics of various indicators of the spread of SARS-CoV 2 were associated with the difference in the daily schedule on weekdays and weekends. The results of the calculations showed that the daily schedules of people strongly influence the spread of SARS-CoV 2. Under assumptions of the model, the results show that for the more contagious “rapid” strains of SARS-CoV 2 (omicron), immunocompetent people become a significant source of infection. For the less contagious “slow strains” (alpha) of SARS-CoV 2, the most active source of infection is immunocompromised individuals (pregnant women). The more contagious, or “fast” strain of the SARS-CoV 2 virus (omicron), spreads faster in public transport. For less contagious, or “slow” strains of the virus (alpha), the greatest infection occurs due to work and educational contacts.

## 1. Introduction

Viral infections have caused and continue to cause the largest epidemics humanity has ever encountered [1,2,3]. One of the greatest dangers posed by epidemics is their ability to mutate and give rise to new species and strains of viruses to which humans have no immunity. This includes SARS-CoV-2, influenza viruses, and others. The problem of preventing the spread of viral infections is associated with several factors, including the presence of an incubation period and asymptomatic individuals. These factors make it impossible to detect such individuals without special diagnostics, and they, in turn, can infect others. Thus, there is a constantly active dynamic source of infection. Additionally, each viral infection has its own parameters of disease progression [4,5]. This, in turn, makes it impossible to establish universal rules for preventing an epidemic.

Vaccination is known to be one of the most effective methods of preventing the onset of an epidemic. However, it is only effective against known viruses, and in the case of new viruses, there is no vaccine available. Therefore, the most pressing task for humanity is to develop effective measures and policies that can prevent the onset of an epidemic and halt the spread of the virus at an early stage. Such measures should be implemented at various levels: cities, regions, countries, and globally. The most common preventive measures against epidemics include wearing masks, restricting people’s mobility, implementing various degrees of quarantine, rapid and comprehensive diagnostics, and utilizing information technologies for contact tracing, among others. At the national and global levels, measures such as border closures, vaccination, and restrictions on travel between countries and regions are being implemented [6,7]. However, there is still no clear management for preventing the spread of epidemics caused by new virus strains. The evidence for this is the rapid spread and global impact of the recent COVID-19 pandemic [1].

It is worth noting that current research focuses on various aspects of viral diseases. Some scientists concentrate on studying the viral infection itself [8]. Certain models simulate a virtual environment and the spread of viral infection within it [9]. Some researchers analyze the statistics and dynamics of infection spread and make predictions based on them for future trends [10,11,12,13]. However, this approach does not allow for predicting the dynamics of infection spread for new prevention strategies or new strains. This is because machine learning methods interpolate well within the training dataset, but they cannot forecast situations or analyze new factors that are absent from the training data.

Another approach is to create a multi agent system that simulates the functioning of objects that spread viral infections. Such models make it possible to simulate human contacts at the micro- and macro-level as realistically as possible, simulate virus transmission and analyze statistical parameters, and conduct computer experiments to analyze the effectiveness of various epidemic prevention strategies [14,15,16,17,18,19,20,21,22,23,24,25,26,27,28,29,30,31,32,33,34,35,36,37,38].

Based on the above, it is currently relevant to develop a mathematical model and create a simulator program based on it to simulate the geo-spatial spread of viral infections at the city, regional, national, and global levels. Generally, the following requirements can be outlined for such a mathematical model:

1. It must be adequate and accurate;
2. It should be relatively straightforward;
3. It should be able to accommodate new factors;
4. It should consider the characteristics of viral infections;
5. It should realistically interpret human life and contacts:
  - Taking into account the age and immune characteristics of individuals;
  - Considering the specific daily routines of residents;
6. It should display results on a geographical map;
7. It should have fast calculations to enable a large number of computer experiments. It is necessary for an analysis that can handle large amounts of sophisticated data to provide reliable predictions for epidemic prevention strategies. These requirements are met by the GeoCity model and were developed by us in Ref. [39].

Therefore, the main objective of this study is to adapt, implement, and verify the adequacy of the multi-agent mathematical model GeoCity for modeling the geo-spatial spread of three different strains of the SARS-CoV-2 virus using the example of the city of Lviv, Ukraine, taking into account the peculiarities of the disease progression in different population groups. This means that we investigate only the course of the epidemic without using any measures to prevent it, such as medical facilities, isolation, traffic stops, etc. If the adequacy of the model is confirmed, it can be used to determine optimal epidemic prevention strategies using neural networks with reinforcement. The parallelization of this model for multiple cities will make it possible to model and formulate measures to prevent the spread of the epidemic within the macro level of the country and the world as a whole.

## 2. Literature Review

As mentioned above, there is no single approach to modeling the spread of new virus strains. There are numerous review papers that analyze and compare different approaches, their advantages, and disadvantages. In particular, Ref. [14] presents and discusses the main approaches used for monitoring and modeling the dynamics of infectious diseases. The fundamental concepts underlying their implementation and practice are explored, and an annotated list of representative works is provided for each category. Ref. [15] analyzes the methods of reporting systematic reviews and meta-analyses in evaluating healthcare interventions. The PRISMA methodology, consisting of a 27-item checklist and a 4-phase flowchart, is discussed in detail.

As demonstrated in these works, the most appropriate methods for analyzing the spread of new virus species and strains, as well as evaluating new strategies and policies, are those based on multi-agent systems and cellular automata. They allow for simulating real human behavior and virus transmission in a virtual world, enabling virtual experiments. These models can be divided into two levels: pedestrian level and macro level (cities, states, and the world).

### 2.1. Pedestrian-Level Models

These models simulate human behavior in relatively small, enclosed spaces and investigate virus transmission through human contact. For example, Ref. [16] presents a pedestrian-based epidemic spread model capable of simulating the risks of disease transmission in indoor environments during human social activities. In Ref. [17], the degree of contact between pedestrians at different pedestrian concentrations was determined through slow-motion recordings, serving as the basis for determining the probability of virus transmission. A microscopic pedestrian simulation model was considered in Ref. [18], where each agent represents an individual. Quantitative assessments of pedestrian infection risks were conducted in Ref. [19]. The infection of airplane passengers was studied in Ref. [20], while the clinical study of patient infection in a hospital was presented in Ref. [21]. These studies allow for establishing functional dependencies for the infection of individuals in confined spaces, providing a basis for constructing macro-level virus spread models.

### 2.2. Macro-Level Models

These models can be further divided into three groups: models based on static and movable cellular automata, as well as multi-agent systems.

#### 2.2.1. Static Cellular Automata

Works that investigate mathematical models of static cellular automata include Ref. [22], where the developed model analyzed the impact of social isolation characteristics on population dynamics, simulated the number of COVID-19 deaths due to the absence of healthcare infrastructure, and studied the effect of healthcare system actions on the crisis in Brazil. Effective modeling of the dynamics of infectious disease spread in spatially constrained environments is presented in Ref. [23], which examines the interaction between humans and pathogens using the example of tuberculosis transmission. In Ref. [24], a SLIRDS model (Susceptible-Latent-Infected-Recovered-Dead-Susceptible) was created based on cellular automata theory and compartmental models, which can better reflect the actual infectious process of infectious diseases. Using the SLIRDS model, the complex process of pandemic influenza A (H1N1) spread was simulated. The use of cellular automata (CA) for creating an epidemic computational model of virus spread in supermarkets under various conditions is analyzed in Ref. [25], employing an approach that has not been used before. In Ref. [26], a 2D cellular automaton based on the SI epidemic model is proposed to determine the most desirable testing frequency and the optimal size of random traces in local urban environments for diagnosing SARS-CoV-2 and isolating infected individuals.

#### 2.2.2. Movable Cellular Automata

The application of movable cellular automata for predicting the spread of the SARS-CoV-2 virus in countries such as Turkey, Ukraine, Serbia, and Slovakia was conducted in a study [27]. The study proposed a model that allowed for the comparison of simulation models with real regions and countries in terms of time and scale. The accuracy of this model was demonstrated, and an analysis of different strategies for preventing viral infection spread was performed. In another study [28], spatial factors influencing physical distancing and their impact on the transmission of the SARS-CoV-2 virus were investigated by integrating pedestrian dynamics with a modified susceptible-infectious-recovered model. The generalization of a mathematical model for determining the temporal evolution of the population in infectious diseases of the COVID-19 pandemic, based on the Kermack–McKendrick model, was explored in a study [29]. Ref. [30] proposes a new optimization algorithm for COVID-19 (CVA) that covers almost all possible optimization problem domains. It also models the process of coronavirus spread in multiple countries worldwide and the spread of the coronavirus as an optimization problem to minimize the number of countries infected with SARS-CoV-2. Three scenarios for solving the optimization problem using the most effective distribution factors were proposed. In Ref. [31], a stochastic structure based on movable agents was developed, allowing for the modeling of clinical and social heterogeneity. All these models are more adequate for real social systems, although they are significantly slower.

#### 2.2.3. Multi-Agent Systems

In contrast to movable cellular automata, which resemble the movement of molecules in a closed environment, multi-agent systems operating according to a daily schedule provide a more realistic simulation of the functioning of a city, region, or country as a whole. Moreover, they are considerably faster than movable cellular automata. This approach was demonstrated in Ref. [32], which described the development and implementation of an agent-based epidemiological modeling system. A similar model allows for the modeling of the dynamics of SARS-CoV-2 spread among city residents and is described in Ref. [33]. In Ref. [34], the modeling results of COVID-19 spread in a limited region with specific demographic characteristics and social relations were presented. This study aims to explore the impact of preventive methods, such as quarantine, social distancing, and reduction of mass transportation, on the spread of epidemics. The adequacy and speed of these models enable their use for automatically determining optimal pandemic prevention strategies and optimal government policies, as presented in Ref. [35]. This article investigates how reinforcement learning (RL) can be used to optimize a mitigation policy that minimizes the economic impact without overwhelming hospital capacity. In Ref. [36], multi-agent modeling (MAS) is used to examine the optimal strategy for preventing the spread of COVID-19 in Japan. Different recommendations for optimal strategies to suppress the pandemic by combining reinforcement learning and MAS were also proposed. This study highlights the potential of MAS in developing infection prevention strategies.

#### 2.2.4. Geospatial Models

Despite the power and capabilities of these models, one drawback is the lack of visualizing results on a geographic map. Such a representation of results would allow for a visual assessment of the scale and dynamics of infection spread. There is a series of works that utilize cellular automata for these purposes, including Ref. [37], which presents the Flu And Coronavirus Simulator (FACS), a modeling tool that simulates virus spread at a subnational level, including geospatial data sources to identify buildings and residential areas within a region. FACS enables modeling the spread of COVID-19 at the local level and provides estimates of infection spread and hospital admissions for different scenarios. The geospatial spread of SARS-CoV-2 on Chikungunya Island was investigated in Ref. [38].

## 3. Materials and Methods

To eliminate ambiguities in the terminology of multi-agent systems (MAS) theory and medicine, we introduce the following definitions:

- Respondent in medicine is defined as an agent in MAS;
- Viral infection in medicine refers to a set of rules and properties in MAS.

In this work, we have expanded the functionality of the GeoCity model (1) proposed by us in Ref. [39]. The model is based on a dynamic multi-agent system that incorporates individual health characteristics of agents (H) and rules for the spread of the SARS-CoV-2 virus (V).

(1) $$\mathrm{GeoCity}={\left\{G,T,\;A,\;R,H,V\right\}}_{\mathrm{city}}$$

Here, G represents a set of geo-objects, T denotes transport, A is the list of agents residing in the city, R represents daily schedule rules for agents, and there is a set of data describing the interaction processes. This includes the health status of an agent (H) and the rules for the spread of infection upon contact with infected and healthy agents (V).

Similar to the work in Ref. [39], we considered that agents can spend their working hours (depending on their age and their profession) in the following facilities: childcare centers, schools, universities, offices, transport drivers, sellers, teacher in the school, etc. For each agent, taking into account his age, a unique daily hourly schedule was constructed for both weekdays and weekends. The age of respondents also affects their medical indicators, such as contagious level and immune system condition.

The model itself consists of three stages. The first stage involves initializing the virtual city and its population, as described in Ref. [39]. The second stage consists of the initialization of parameters related to the viral infection. The final stage is the simulation of the city’s functioning and the spread of the viral infection, considering hourly time intervals and accounting for working days and weekends.

### 3.1. Stage 1: Model Initialization

The model was initialized following a similar approach as in Ref. [39]. The main differences were related to agent generation. Specifically, we took into account the following age groups: Children (0–6), Pupil (7–17), Young (17–44), Adult (45–60), and Elderly (60+). The age of each person within a group was randomly generated within the corresponding age range. Depending on their age, individuals could have different professions. For example, children aged 0 to 4 could either stay at home or attend a childcare center. An adult living with a minor child was assigned the profession of “mother”. Children aged 4 to 14 were considered pupils. People aged 15 to 24 could be university students or employed. Individuals over the age of 60 were considered retirees. All other individuals between the ages of 25 and 59, as well as young adults of student age who were not students, could work in professions such as office worker, store clerk, salesperson, or public transportation driver. Agents without a profession were marked as unemployed.

To simulate the spread of the virus, it was necessary to indicate the immune system state $h_{i}$ of each individual $a_{i}$ during the model initialization stage. This state was described using the following parameters:

- Health status ($S_{i}=\left[“S”,“E”,“I”,“R”\left(“D”\right)\right]$): S—susceptible, E—exposed, I—infected, R—recovered, or D—deceased (at the initial stage, all individuals were healthy (S));
- Immunity status ($im_{i}=\left[0,\;1\right]$): 0 represents no immunity to the infection, and 1 represents 100% immunity (recovered individuals);
- Contagiousness level ($con_{i}=\left[0,\;10\right]$): determines the infectiousness of a specific infected person, indicating how likely they are to infect others;
- Asymptomatic status ($sym_{i}=True/False$): indicates whether a person will exhibit symptoms or be asymptomatic when infected. The course of the disease in people with asymptomatic status is the same as in the other person. However, it occurs without external manifestations of the illness. This means that they cannot be identified as sick and cannot be directed to compulsory hospitalization or home quarantine;
- Contagiousness level on a specific day t—$con_{i}^{t}$;
- Infection time, $t_{i}^{inf}$ (days);
- The number of people infected by the agent, $R_{i}$.

(2) $$H={\left\{h_{i}\right\}}_{i=1,P}={\left\{S_{i},\;{im}_{i}\;{con}_{i},\;{sym}_{i},\;{con}_{i}^{t},t_{i}^{inf},\;R_{i}\right\}}_{i=1,P}$$

Here, i represents the agent identifier and P denotes the number of agents.

These characteristics depend on individual parameters, such as age and pregnancy status in women. Pregnancy status is randomly determined for adult individuals based on statistical data [40].

### 3.2. Stage 2: Initialization of Rules for the Course of Viral Infection

By these rules, we understand how the agents’ health parameters $h_{i}$ will change during interaction with other agents and throughout the disease. Unlike other model attributes, these rules are described using functional dependencies under the SEIR(D) approach.

(3) $$V=\left\{con\left(t_{st}\right),{St}_{inf}\left(P_{inf}\right),{St}_{R\left(D\right)}\left(t_{st},\;t_{i}\right)\right\},$$

Here, $con\left(t_{st}\right)$ is the function that determines the contagiousness level of the infected agent, $St_{inf}(P_{inf})$ is the function that determines the change of state for a susceptible agent after contact with an infected agent, $P_{inf}$ is the function representing the probabilities of infection, and ${St}_{R\left(D\right)}\left(t_{st},\;t_{i}\right)$ is the function that determines whether an infected agent will recover or die after experiencing the illness. Let us consider each of these functions in turn.

#### 3.2.1. Contagiousness

According to the classical SEIR(D) model, agents can be in four stages: “S”—susceptible, “E”—exposed, “I”—infected, and “R” (“D”)—recovered or deceased. The lifecycle of a real agent consists of the following steps. A susceptible person can interact with an infected agent, and, with some probability, the susceptible agent can be infected. Upon infection, the agent transitions to the “E” state, where it does not exhibit symptoms but can infect others. After a certain time, $t_{i}$, the agent transitions to the “I” state, indicating the presence of symptoms. Eventually, after time $t_{r}$, the agent either recovers (“R”) or dies (“D”). Studies [36] have shown that for real illnesses, another dependency of infectivity or contagiousness is observed. After infection, an agent is initially asymptomatic and cannot infect another agent. After some time, $t_{c}<t_{i}$, the agent becomes capable of infecting others with varying probabilities. After time $t_{i}$, the agent develops symptoms of the illness. These times vary for different types of illnesses. During the lifecycle, the contagiousness of the agent changes, reaching maximum values at the end of the “E” state and the beginning of the “I” state. We propose describing the level of contagiousness using the L-R membership function from fuzzy logic, which is used to describe natural processes.

(4) $$con\left(t_{st}\right)=\left\{\begin{array}{cc} t_{st}\leq t_{i} & \max\left(0,Real\left(\sqrt[{2}]{1-{\left(\frac{t_{i}-t_{ts}}{a}\right)}^{2}}\right)\right)\\ t_{st}\geq t_{i} & e^{-{\left|\frac{t_{st}-t_{i}}{b}\right|}^{3}} \end{array}\right.,$$

Here, $t_{st}$ this is the time that has passed since the moment of infection of the agent, and a and b are empirical parameters.

**Figure 1:**
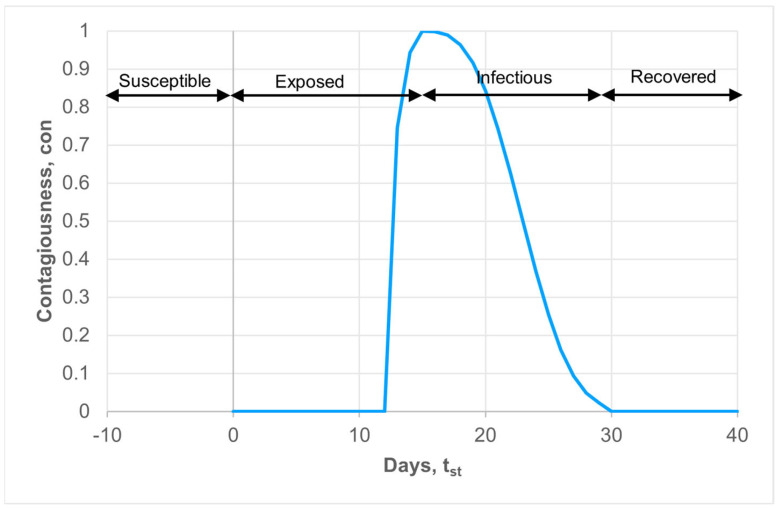
Contagiousness of the infected agent dependency on time.

For COVID-19, the duration of the “E” state without contagiousness strongly depends on the virus strain. Additionally, it can vary significantly within a particular strain based on individual characteristics and the date. For example, for the alpha strain, it can range from 4.5 to 22 days. To ensure the adequacy of the model, we will assume 12 days for this period [41]. After this period, the agent can infect others for 2 days, leading to $t_{i}$ = 14 days. The symptomatic phase begins after this period and can be described by Equation (4) with a=5 and b=9 (see Figure 1).

In this approach, we can merge the “E” and “I” state. It means that the agent is infected with different levels of contagiousness.

#### 3.2.2. Infecting

As previously mentioned, agents interact in various locations such as workplaces, supermarkets, and transportation, as well as within their families throughout the day. The are two ways to simulate viruses spread in these objects:

1. The multi-agent approach [36], which needs a lot of computer time;
2. Probability theory [42]: if a susceptible person is present in a location where infectious individuals also reside on the same day, the probability of infection can be calculated as follows

(5) $$P_{inf}=\left(\frac{LS_{s}}{OD_{loc}}\times \frac{LS_{i}}{A_{con}}\right)\times \frac{IR}{360}\times M$$

Here, $P_{inf}$ presents the probability of a susceptible person becoming infected. $LS_{s}$ is the duration of stay of the susceptible person in minutes, $LS_{i}$ is the duration of stay of the infectious person in minutes, ${OD}_{loc}$ is the opening duration of the location on that particular day, $A_{con}$ is the area of the location in square meters, IR is the infection rate of viruses (e.g., 0.07), the empirical value, and M is a static contact rate multiplier with a default value of 1.

It should be noted that in our approach, $LS_{s}$, $LS_{i}$, and $OD_{loc}$ are approximately equal for each area. Obtaining precise information about the area of each location in a real city is challenging. Instead of the room area, we suggest using the number of contacts in the room. If, on average, one agent can come into contact with n agents in a specific location during one iteration, then the probability of infection will be inversely proportional to the total number if there are more than n agents in the workplace, transportation, or supermarket. This is because an infected agent cannot contact all of them. Conversely, if there are only two agents in the room, the contact time and the probability of infection will increase accordingly. Therefore, we propose modifying Formula (5) as follows:

(6) $$P_{inf}=LS_{i}\times \frac{IR}{360}\times N_{inf}\times \frac{n}{N_{obj}}\times \sum_{a=1}^{N_{inf}}con_{a}\times (1-imm).$$

Here, $N_{obj}$ represents the number of agents in a specific location, $con_{a}$ is the level of contagiousness of agent a, and imm is the level of immunity of a susceptible agent (an empirical value that can be obtained from clinical investigations [0—no immunity, 1—100% immunity to illness]). If there are $N_{inf}$ infected agents in the same location, the probability should be proportionally increased.

Based on the obtained probability of infection, a change in the state of a susceptible agent is determined as:

(7) $${St}_{inf}\left(P_{inf}\right)=\left\{\begin{array}{cc} I & \;x<P_{inf}\\ S & x\geq P_{inf}\; \end{array}\right.$$

Here, x ∈ [0, 1] represents a random number.

#### 3.2.3. Recovering

An infected agent can either recover or die, depending on its probability of death. Recovery occurs when an infected agent becomes ill, and its infection level falls below a certain small threshold:

(8) $${St}_{R\left(D\right)}\left(t_{st},\;t_{i}\right)={\left(\begin{array}{cc} R & \;x\geq P_{death}\\ D & x<P_{death}\; \end{array}\right)}_{t_{st}>t_{i}\;and\;con\left(t_{st}\right)<\alpha }$$

Here, α represents the threshold level of contagiousness.

### 3.3. Stage 3: Simulating the Functioning of the City and Virus Spread

This stage consists of the following steps.

#### 3.3.1. Step 1: Initial Infection

Before starting the simulation, it is necessary to define the source of infection. It should be noted that the model is probabilistic and heavily dependent on the initially infected individual, including their interactions, cohabitation, and daily schedule. Therefore, during the initial stages of modeling, significant fluctuations in all statistical parameters are possible. To reduce the impact of stochasticity and facilitate convergence to the laws of large numbers, several infected individuals should be used as the source of infection. In our calculations, we considered a group of 10 infected individuals.

#### 3.3.2. Step 2: Determining the Health Status of Individuals at the Beginning of the Day

At the start of the working day, the infection duration for each infected individual is increased by one day. Their personal level of contagiousness at the beginning of the day is also recalculated based on the number of days they have been infected ($t_{ts}$) according to Equation (2). For non-infected individuals, $con_{i}^{ts}=0$.

#### 3.3.3. Step 3: Simulation of the Working Day

During this stage, based on the schedule (considering working days and weekends), the location of each individual is determined hourly. The objects $\left(v\;\subset \;J\right)$ where infected individuals are present are identified for each hour. The number of infected individuals present in each object $\left(N_{inf}^{v}\right)$ is determined. For each healthy individual located near infected individuals, the probability of infection is calculated using Formula (6). The status of healthy individuals (S) is then changed to infected (I) according to Equation (7).

According to Formula (6), we cannot clearly determine which of the sick people identified the healthy one. Therefore, in case of a change in the health status of a healthy individual, the coefficient $R_{i}$ is increased symmetrically for each infected individual to the level of $con_{i}^{ts}$.

#### 3.3.4. Step 4: End of the Working Day

At the end of the simulation of the working day, the possibility of recovery or death is determined for all infected individuals according to Formula (8). Statistical data is analyzed and saved, including the calculation of the basic reproductive number, which is defined as an average number of infected agents by the sick agent:

$$R_{0}={\bar{{(R}_{j})}}_{R_{j}>0}$$

The simulation continues until there are no infected individuals left or until it is forcibly stopped.

## 4. Results

### 4.1. Population Generation

The purpose of this paper was to test the relevance (adequacy) of the proposed model for simulating three variants of SARS-CoV-2, namely Alpha, Delta, and Omicron. In this analysis, we will not consider any means of preventing the spread of these viral infections by the government. In other words, we will try to simulate the spread of infections without any human or governmental response, which will allow us to observe how the infection would develop on its own and what consequences it would lead to.

Each of these variants of coronavirus strains possesses distinct characteristics. There are eight major variants of the novel coronavirus SARS-CoV-2 that are of concern to medical professionals at the moment. In the summer of 2021, the World Health Organization decided to designate SARS-CoV-2 variants using letters from the Greek alphabet instead of using the names of countries where they were detected. It all started with the variant L, which was detected in Wuhan, China, in December 2019. The most prevalent variants on the European continent, including Ukraine, are Alpha, Delta, and Omicron. It is these particular variants that we have taken into account in this study.

The “Alpha” variant-B.1.1.7 (Kent or British variant) has specific symptoms: cough, severe fatigue, sore throat, and muscle pain, with loss of smell and taste occurring rarely. The “Alpha” variant spreads more quickly compared to the original Chinese variant.

The “Delta” variant-B.1.617.2 (Indian variant) causes leading symptoms such as headache, sore throat, malaise, and fever. Patients infected with the Delta variant mention cough and loss of smell much less frequently. According to Indian doctors, people infected with the Delta variant more often experience hearing loss, severe abdominal pain, and nausea. They also complained of dryness in the mouth, loss of appetite, and joint pain. In most cases, these patients were hospitalized more frequently, requiring oxygen therapy and suffering from the development of complications.

The “Omicron” variant-B.1.1.529 (South African variant) is highly transmissible. It generally has a milder course compared to the Delta variant, with symptoms such as sore throat, malaise, and slight temperature elevation. Patients with the Omicron variant require professional medical assistance much less frequently.

“Omicron” is capable of increasing reinfection rates by eight times. It reduces the effectiveness of the Pfizer vaccine in preventing hospitalization after two doses to 70%.

**Figure 2:**
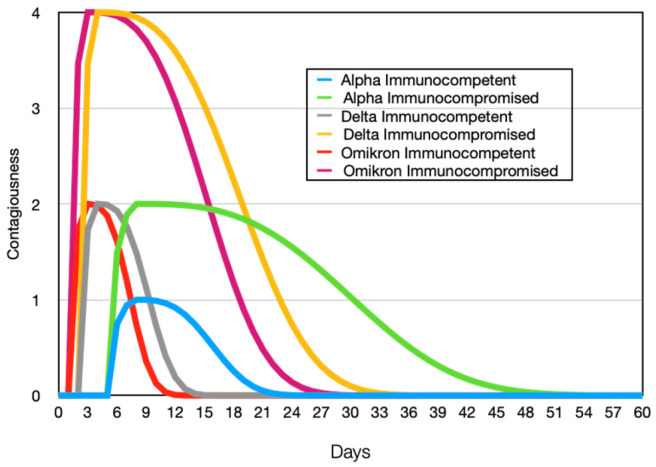
Contagiousness (12) of infected agent dependency on time for different stamp of viruses: Alpha $t_{i}=15,\;a=3,\;b=9$, Delta $t_{i}=4,\;a=2,\;b=6$, Omikron $t_{i}=3,\;a=2,\;b=5$.

Mathematically, the level of contagion depending on the duration of infection can be described using the following graphs (Figure 2).

So, as can be seen from the contagion graphs, the Omicron and Delta variants have the shortest incubation period and are characterized by a rapid course of the disease. In contrast, the Alpha strain has a long incubation period and a prolonged period of illness, during which infected individuals can transmit the infection to others. The graphs clearly show that considering contagion leads to both a higher level of infectiousness in individuals and a longer period of potential transmission to others.

It is believed that the immune system status of respondents affects the contagiousness. According to statistical data [43,44], all respondents are divided into immunocompetent (55%) and immunocompromised (45%) individuals. The group of physiologically immunocompetent individuals includes 50% of people aged 18 to 65 years who harmoniously respond to contact with infectious agents and recover quickly. This is the majority of the population.

This group also includes 5% of people who have a very well-developed genetically based anti-infectious immunity. They can carry various strains of coronavirus asymptomatically, as well as other infections.

The group of immunocompromised individuals includes physiological and pathological subgroups. Physiological immunocompromised subgroups include children (10%) and the elderly 65+ (10%), who are more susceptible to infectious diseases due to age-related peculiarities of immune response to infections. In addition, the incubation period is 2–3 days longer in children and 1–2 days longer in the elderly compared to immunocompetent individuals. Pregnant women (5%) are also included in this physiological subgroup of immunocompromised individuals.

Pathologically immunocompromised individuals include those with genetic immunodeficiencies and people with immune disorders. Up to 1% of people may have significant genetic defects in the immune system and therefore cannot fight off many infections, including coronavirus infection. It has been established that up to 19% of respondents have acquired immune system disorders as a result of immune system dysfunction, leading to chronic diseases (diabetes, hypertension, kidney diseases, etc.), long-term use of immunosuppressive drugs (oncological, autoimmune diseases, post-transplantation), or an unhealthy lifestyle (smoking, obesity, etc.).

### 4.2. City Simulation

**Figure 3:**
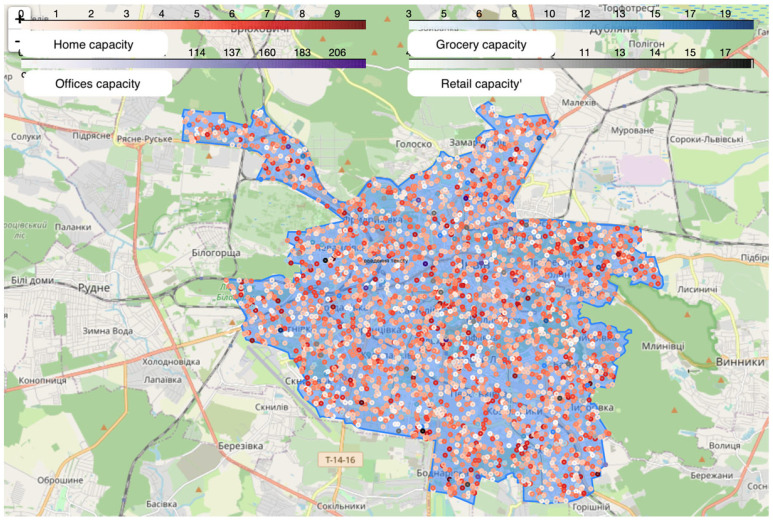
Simulated city Lviv, Ukraine. The circles represent the agent’s possible location (Home, Work, Grocery, etc.). The color determines the maximum number of agents that can be in this object. Blue shape—the investigated territory.

The spread of these coronavirus variants was examined through a simulation of Lviv, Ukraine. Lviv is the largest regional center in western Ukraine, with a population of about 700,000. The city has a circular shape for its center, while the city borders have a complex structure with multiple branches (Figure 3).

The simulation was conducted with the following parameters: 10,000 agents, 3000 homes, 100 grocery stores, 3 preschools, 7 schools, 2 universities, 50 retail stores, 100 transportation hubs, and 50 office buildings. According to statistics [40], only 20% of Ukrainians own a car, which means that 80% of the population use public transport or walk. In our simulation, 50% of the agents use public transport, while 30% are pedestrians.

According to statistical data [40], 60% of children attend preschool, and students make up 80% of the student-age category. Additionally, 6% of people are unemployed. We assume that one family member visits a supermarket near their place of residence after work. Individuals who do not work (mothers, unemployed) and stay at home during the day, on average, visit 2–3 supermarkets and 1 clothing store.

The statistics of job positions obtained after the city simulation are presented in Table 1.

As mentioned above, each individual is assigned a job position. The distribution of job positions and age groups obtained is presented in Table 2.

As seen from the table, the largest number consists of office workers. Young individuals of student age are not only students but also work in various professions. It can also be observed that the number of individuals taking care of children and unemployed middle-aged agents is approximately the same.

### 4.3. Modeling the Spatial Spread of the Alpha Variant Virus

**Figure 4:**
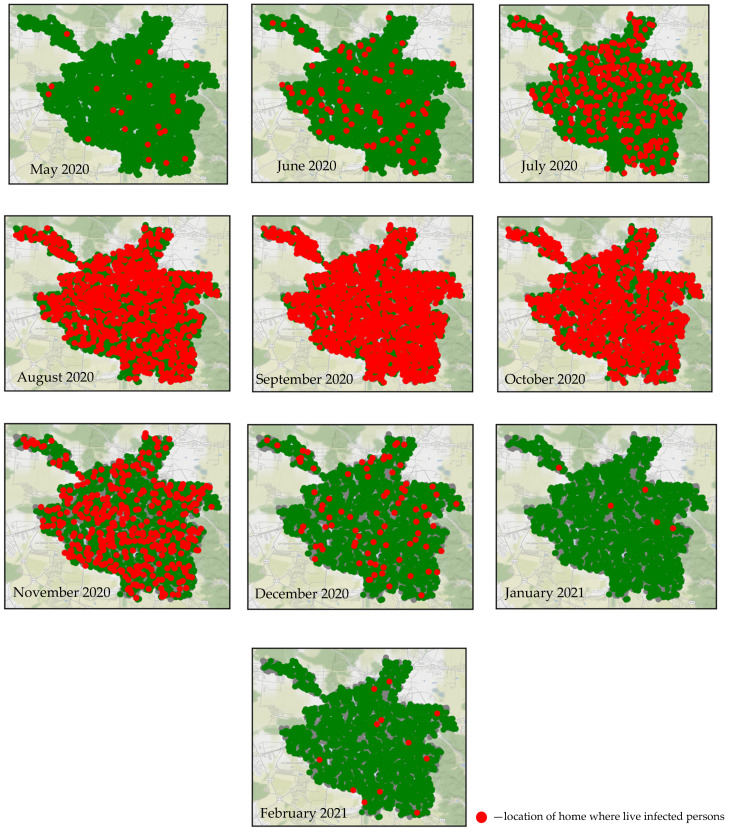
Simulation of residences location in which infectious agents live during the epidemic from 20 March 2020 to 22 February 2021 in Lviv city.

Figure 4 presents the simulated monthly dynamics of the spread of the coronavirus infection throughout the entire epidemic process. Infected individuals’ houses are marked with red dots, uninfected ones with green, and gray dots represent those who have recovered.

As seen from the figures, there is no observed epidemic in the first month. The following two months witness a sharp increase in the number of infected individuals. Then, a rapid decline in the epidemic is observed. Additionally, from the figures, it is evident that a large number of residential buildings remained uninfected. The total duration of the epidemic lasted for four months. Moreover, it can be observed that the infection spreads evenly across the entire city, with no distinct clusters of infection.

### 4.4. Modeling of the Alpha, Delta, and Omega Viruses’ Spread Dynamics

**Figure 5:**
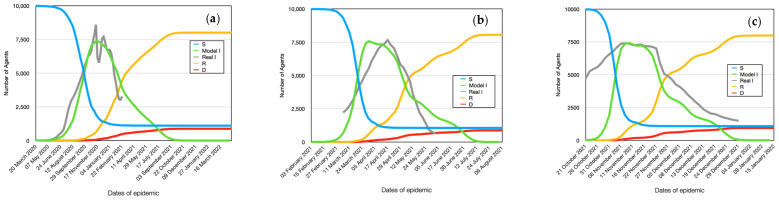
Dynamics of the number of infected, healthy, recovered, and deceased individuals in the simulation. Alpha (**a**), Delta (**b**), and Omicron (**c**).

**Figure 6:**
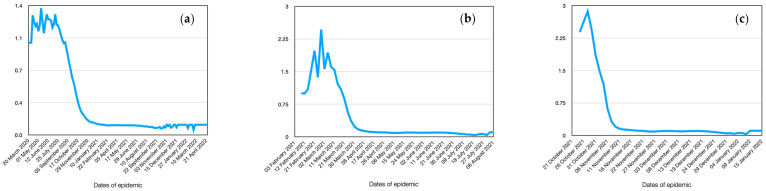
Dynamics of the base reproductive number $R_{0}$ in the simulation. Alpha (**a**), Delta (**b**), and Omicron (**c**).

A more visual representation of the infection dynamics in comparison with real cases for the Alpha stamp is presented in Figure 5a. It should be noted that in order to compare the obtained results of the model, the real data were reduced to the scale of the multi-agent system. The first outbreak of coronavirus of this strain was observed in the period from 20 March 2020 to 22 February 2021. Subsequently, the next peak was observed, associated with the appearance of a new strain of the virus. As can be seen from the figure, the obtained results quite well repeat the main trends observed in clinical cases. As seen from the figure, the peak of the epidemic occurred on the 40th–50th day (considering a 5-day incubation period, it corresponds to the 35th–45th day from the onset of the first symptoms). Additionally, the graph shows slight fluctuations in the dynamics trend, which can be attributed to different daily schedules of agents during weekdays and weekends. The figure also indicates that approximately 20% of agents remained uninfected. The dynamics of the reproduction number (Figure 6a) is interesting. It can be observed that this indicator fluctuates significantly during the initial stage of the epidemic, reaching a value of 6a. Gradually, by the 40th day, it stabilizes at around 3.8, which aligns well with the experimental data [40].

According to the geospatial modeling of the spread of Delta coronavirus strain, it spreads uniformly throughout the city, similar to the previous case, but at a much faster rate. This can be observed from the graph depicting the dynamics of the number of infected individuals (Figure 5b). As seen, the peak of the epidemic occurs on the 18th to 20th day after the onset of infection. The number of uninfected individuals remains approximately at the same level. Weekly fluctuations in the dynamics of infection spread to become more pronounced.

The reproductive number is slightly higher, reaching 6.0 (Figure 6b). Within a shorter period of time, infected individuals have the opportunity to come into contact with slightly fewer agents in their surroundings. Therefore, the increase in the reproductive number is specifically associated with the increased contagiousness of the virus itself.

The simulation results agree quite well with real clinical cases. It should be noted that the deviation is somewhat larger because in a real case, the strains of two coronaviruses are superimposed. In addition, deviations are associated with the approximation of the model, which cannot account for all factors.

The omega virus strain is the most aggressive in terms of infectivity among people. It is evident that if no preventive measures are taken, within a short period of 10 days, almost all individuals who come into contact with infected individuals will become infected. The only ones not infected will be those who have not crossed paths with the infected individuals (Figure 5c). Thus, on the 10th day, there is a nearly 100% infection rate in the city, leading to a total epidemic. Weekly fluctuations in infections become more pronounced.

As can be seen from the figure, the deviation of model data from real data in this case is greater. This is due to the fact that the disease lasts for a long time, and many different factors have accumulated that were not taken into account in the model. A good agreement with the experimental data and the growth of the accumulated error may indicate the adequacy of the proposed model. Analysis of 3 different types of virus strains allowed us to assess the sensitivity of the model to various factors.

The reproductive number reaches its maximum possible level ($R_{0}\;$= 8.9) (Figure 6c). Over the course of the week, agents recover, and the epidemic declines.

### 4.5. Modeling of Immunocompromised and Immunocompetent Individuals Spread Dynamics

**Figure 7:**
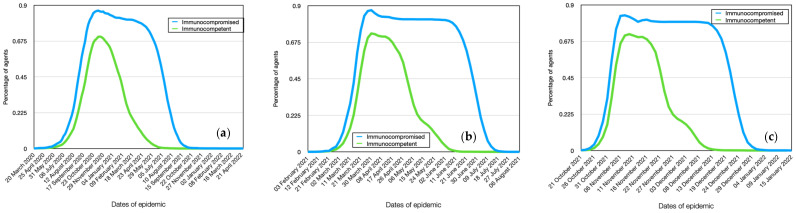
Comparative dynamics of the number of immunocompromised and immunocompetent individuals. Alpha (**a**), Delta (**b**), Omicron (**c**).

According to the simulation results, the dynamics of immunocompromised and immunocompetent individuals for Alpha stamp are also intriguing. Despite the lower overall immunity of immunocompromised individuals to various infections, their relative number affected by the coronavirus infection is only slightly higher than that of immunocompetent individuals (Figure 7a), considering that almost all healthy individuals also get infected. Furthermore, from the graphs, it can be observed that immunocompromised individuals become infected faster than immunocompetent ones, reaching a rate of 86% on the 43rd day from the start of infection. This is particularly evident from Table 3, where 25% of infections among immunocompromised individuals is reached on the 28th day, whereas the same level for immunocompetent individuals is achieved in 32 days, i.e., 4 days later. The 50% infection level among immunocompromised individuals occurs 5 days earlier than in immunocompetent individuals. Thus, the peak of the epidemic for immunocompetent individuals occurs approximately one week later than for immunocompromised individuals. The maximum infection rate for immunocompetent individuals is 75%. Additionally, from the graph, it can be seen that immunocompromised individuals have a significantly longer duration of illness. Moreover, their dynamics show a slight peak on the 65th day, which is due to children having a longer incubation period, thus experiencing the peak of the disease later, as will be shown further.

**Figure 8:**
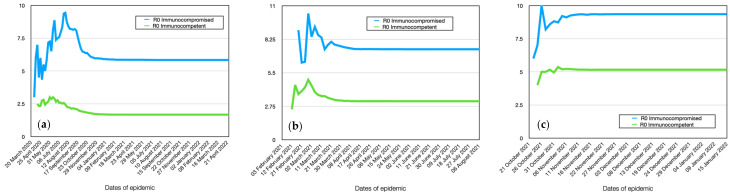
Comparative dynamics of the reproductive number ($R_{0}$) for immunocompromised and immunocompetent individuals in the simulation. Alpha (**a**), Delta (**b**), Omicron (**c**).

As shown in the model, immunocompromised individuals become infected earlier and have a higher level of contagion, and they can indeed infect significantly more healthy agents (Figure 8a). Therefore, their reproductive number ($R_{0}$) is significantly higher ($R_{0}$ = 5.8) compared to immunocompetent individuals ($R_{0}$ = 1.7). Consequently, immunocompromised individuals are much more dangerous for the spread of the epidemic.

The graph of the dynamics of infection for Delta among immunocompromised individuals clearly shows saturation on the 14th to 15th day (Figure 5b). This is also due to the fact that, on the one hand, younger individuals and children, who have a longer incubation period, join the majority of the population, and on the other hand, this population group experiences a significantly longer duration of illness. This means that in the case of such an active virus strain, 80% of these individuals will be infected within the first days of the epidemic. For immunocompetent individuals, this indicator is 70%, and there is no saturation. In other words, after reaching 70%, the epidemic sharply declines. It should be noted that the difference in the reproductive number between these population groups is also significant (Figure 6b). Specifically, for immunocompromised individuals, $R_{0}$ = 7.5, while for immunocompetent individuals, $R_{0}$ = 3.1. The difference in the dynamics of infection between these 2 groups is smaller and amounts to only 2 days, unlike the 5 days for the previous virus strain (Table 4). Therefore, infected immunocompetent individuals start infecting others earlier and become more active sources of transmission compared to the previous case.

**Figure 9:**
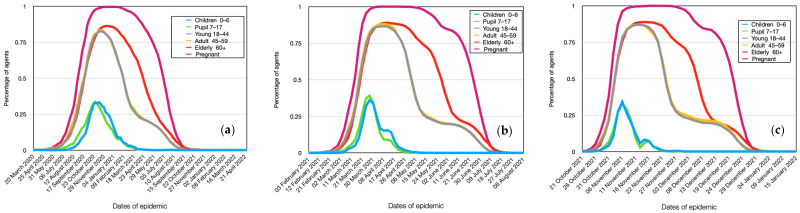
Comparative dynamics of the number of infected individuals in different age groups in the simulation. Alpha (**a**), Delta (**b**), and Omicron (**c**).

**Figure 10:**
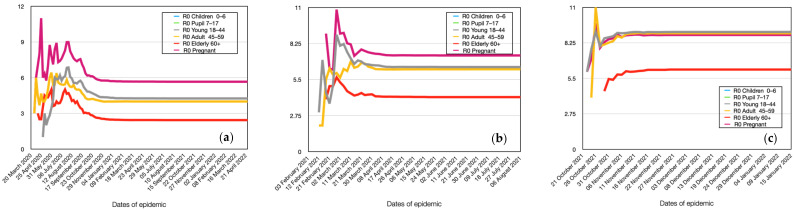
Comparative dynamics of the reproductive number in different age groups in the simulation. Alpha (**a**), Delta (**b**), Omicron (**c**).

**Figure 11:**
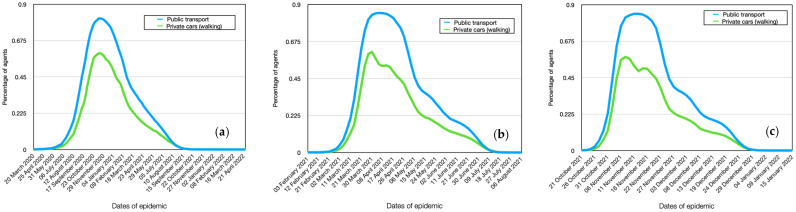
Comparative dynamics of the number of infected individuals depending on transportation in modeling. Alpha (**a**), Delta (**b**), Omicron (**c**).

**Figure 12:**
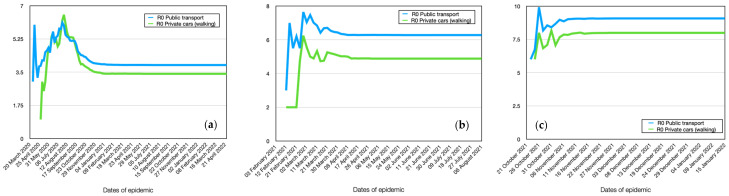
Comparative dynamics of the reproductive number depending on transportation in modeling. Alpha (**a**), Delta (**b**), Omicron (**c**).

The dynamics of all indicators for Omicron in most cases are similar to the Delta coronavirus strain, with differences only in absolute values (Figure 7, Figure 8, Figure 9, Figure 10, Figure 11 and Figure 12, Table 5, Table 6, Table 7, Table 8, Table 9 and Table 10). The following differences should be noted: the difference in infection between immunocompromised and immunocompetent individuals is only 1 day (Table 5). The difference in reproductive numbers for these population groups is at the same level and amounts to 9.3 and 5.1, respectively.

### 4.6. Modeling of Age Groups Spread Dynamics

Of particular interest is the comparison of infection spread among agents in different age groups, taking into account the group of pregnant women. The results for Alpha are presented in Figure 9a. As can be seen from the graph, a distinct group of young people aged 0 to 18 years stands out. As mentioned earlier, they have an incubation period that is 3 days longer. This means that in the case of an active virus, schoolchildren and children attending daycare are more likely to be infected by individuals from other groups in their place of residence rather than within their own group (school, daycare). These children form somewhat closed systems, which results in a significantly lower number of infected children compared to other groups, despite their higher level of contagion.

As mentioned earlier, older individuals have a 2-day longer incubation period. However, this period is not sufficient to exhibit behavior similar to that of young people.

Interesting results were obtained when analyzing the reproductive number (Figure 10a). As seen from the figure, pregnant women infect the highest number of agents. This is due to their high level of contagion and having a similar work week as ordinary people. Despite the higher level of contagion in the elderly, they exhibit lower values of the reproductive number. This is due to their limited social interactions during the epidemic. Agents with normal immune function infect a significantly larger number of healthy individuals due to their active lifestyle. This is observed for young and middle-aged individuals without restrictions. It should also be noted that preschoolers and schoolchildren infect fewer other agents due to their longer incubation period. By the time they can infect others, the agents around them are already either sick or immune. Therefore, this group of agents is a consequence, rather than an initiator, of the epidemic.

As can be seen from the obtained plots, the main difference in the dynamics of infection among age groups of the population for Delta (Figure 9b) is the presence of clear saturation for certain groups, such as pregnant women. This means that, accordingly, the model results show that such an active virus strain is capable of simultaneously infecting 100% of these individuals. Similarly, the group of preschoolers and schoolchildren stands out, as they get infected later and experience significantly fewer cases of illness compared to the general population.

The dynamics of the reproductive number remain similar to the previous virus strain (Figure 10b). This means that regardless of the virus strain, the individual infectivity of a respondent no longer plays a significant role. What matters is the circle of contacts and interactions of the infected respondent.

There is also a difference in the reproductive number across age groups for Omicron (Figure 9c). Specifically, the reproductive number for young and middle-aged individuals is approximately the same as for pregnant women. This may be due to the fact that the activity of this virus strain is a more significant factor than the immunity and personal infectivity of an individual. The trends in the corresponding changes of various parameters, depending on the virus strain, confirm the robustness and adequacy of our model.

### 4.7. Modeling Spread Dynamics Dependence on Transport

The results of the calculations show that different behaviors are observed among agents depending on their mode of transportation: public transport or private cars (walking). The infection dynamics for Alpha for these individuals are nearly identical. This is because the highest number of contacts occurs on workdays. However, agents using public transport infect a larger number of other agents ($R_{0}$ = 3.9 for public transport, $R_{0}$ = 3.4 for private cars), as reflected in the reproductive number (Figure 11a and Figure 12b). It should be noted that agents using public transport become infected on average 3–5 days earlier than those using private cars or walking (Table 6). There is also a significant difference in the maximum number of agents infected. In public transport, 81% of agents get infected, while those using private cars (or walking) account for 60%, which is 21% less.

**Table 6:** Number of days to reach the specified infection level for the Alpha variant depends on the type of transport.

	Public Transport	Private Cars (Walking)	Difference
25%	30	33	3
50%	34	39	5
75%	40		

**Table 7:** The number of days to reach the specified level of infection for the Delta and Omicron variants depends on the type of transport.

	Public Transport	Private Cars (Walking)	Difference
25%	14	15	1
50%	16	17	1
75%	18		

Under assumptions of the model, the results show that the mode of transportation, whether public transport or private car, significantly influences the dynamics of infection for Delta and Omicron (Figure 11b). Weekly fluctuations in the graph of agents using public transport or walking to work are clearly visible.

The delay in infection is only 1 day for booth stamp (Table 7). However, the reproductive number increases for both cases (Figure 12b) ($R_{0}$ = 6.3 for public transport, $R_{0}$ = 4.9 for private cars). The difference between them becomes more significant. This means that agents using public transport become more active sources of infection spread than those using private vehicles.

### 4.8. Modeling Spread Dynamics Dependence on Professions

A similar analysis of simulation results regarding professions (Table 8) shows that the highest level of infection for Alpha is observed in professions such as universities and offices. These institutions have the highest number of contacts between infected and healthy individuals, thus resulting in the highest level of transmission.

**Table 8:** Reproductive number for different professions for the Alpha strain.

Profession	$R_{0}$
University Student	6.4
Offices	4.4
Universities	4.1
Grocery	3.6
Schools	3.5
Retail	3.2
Preschools	3.1
Unemployed	2.3
Domestic	2.0
Transport	2.0

Similarly, to the previous Alpha stamp, the Delta and Omicron virus spreads most actively among university faculty and students (Table 9 and Table 10). The next group to be affected is office workers, followed by schools. The lowest number of infections, in the case of a rapid virus strain, occurs among public transport drivers. This can be explained by the virus being highly active, and agents spend the majority of their time at their workplaces rather than in transportation.

**Table 9:** The reproductive number for different professions/specialties for the Delta variant.

Profession	$R_{0}$
University Student	6.75
Offices	6.64
Schools	6.47
Grocery	5.97
Preschools	5.56
Universities	5.17
Retail	3.90
Unemployed	3.77
Domestic	3.57

**Table 10:** The reproductive number for different specialties for the Omicron.

Profession	$R_{0}$
University Student	11.42
Universities	10.60
Offices	8.93
Preschools	7.92
Retail	7.82
Grocery	7.14
Schools	6.76
Unemployed	5.20
Domestic	4.97

## 5. Discussion

In this paper, we explored the adequacy and accuracy of the GeoCity model (1). Specifically, the accuracy of the model was confirmed by comparing modeled data with real data obtained from statistics. Adequacy was tested on well-known data such as the spread of the virus in transportation and across different population layers. Therefore, our model was able to demonstrate its adequacy and confirm commonly known conclusions. This serves as a scientific basis for applying this model in further research.

In particular, the proposed multi-agent system allows for simulating city functioning and infection spread in a highly realistic manner closely resembling the real world. This enables the investigation of parameters that are impossible to study in real life. At any given time, we have data on each agent, allowing us to analyze agent state changes throughout the simulation. It provides the ability to study emergent effects.

Such a simulator program can be used to determine optimal strategies for preventing viral infection spread. Using this model, various scenarios and prevention policies can be simulated and their effectiveness can be evaluated. These scenarios include isolation, vaccination, medical facility treatments, quarantines, weekend lockdowns, and other control methods. Simulation in a virtual environment and the analysis of results offer a safer and scientifically grounded approach to implementing practical measures for infection prevention. The selection of an optimal infection prevention strategy can be realized using reinforcement neural networks.

Another application of this model is autonomous simulation of individual cities and villages that constitute a country, and their integration into a comprehensive system. This approach would allow modeling viral infection spread across an entire country and the world. The possibility of dividing into autonomous objects would enable parallel computations on computer clusters, significantly speeding up calculation and neural network optimization.

Furthermore, the model possesses the capability to expand and account for various types of viral and bacterial infections, making it adaptable and universal for modeling different epidemics.

An additional notable advantage of this model is the ability to visualize results on a geographical map. This is a significant advantage when modeling macro systems. While we demonstrated this capability at the city level, its accuracy could not be verified. The situation will be quite different when modeling at the macro level.

In conclusion, it can be stated that this foundational model holds promising prospects for further usage and integration into management systems.

It should be noted that the results of the model are subject to some assumptions described in Formulas (4) and (6). Of course, if these assumptions are not true in reality, then the claims may not be true. This can happen if the rules of the spread of the virus change, or the structure of the interaction of agents and their daily schedule changes. In particular, the observance of certain safety measures such as masks, national features, or other measures to prevent the spread of the virus, which are not taken into account in the basic model. However, these formulas can be easily adapted to new realities.

Additionally, a caveat to this research is the unique daily hourly schedule, required to understand how much time is spent inside different workspaces. This might be an opportunity to leverage information from smart sensors that can understand when people spend time inside different types of buildings, which can garner information about their daily schedules more precisely [45]. This can also help understand daily schedules during weekdays and weekends.

## 6. Conclusions

In the study, a modification of the previously developed GeoCity model was proposed by refining the age structure of the population, incorporating individual schedules for weekdays and workdays, and considering individual health characteristics of agents. This allowed for the construction of a more realistic model of city functioning. The developed model enabled the simulation of the spread of three strains of the COVID-19 virus and the analysis of the model’s adequacy in the case of unimpeded virus transmission among city residents.

1. Accordingly, the assumption of the proposed model SARS-CoV-2 primarily spreads through contact in workplaces and public transportation, while school children and preschoolers are the victims rather than the initiators of the epidemic;
2. Fluctuations in the dynamics of different indicators of SARS-CoV-2 transmission are associated with variations in daily schedules between weekdays and weekends. The model simulation shows that the daily schedule of individuals significantly influences the spread of SARS-CoV-2;
3. Under model assumption for more contagious “fast” strains of SARS-CoV-2 (e.g., Omicron), immunocompetent individuals become significant sources of infection. For less contagious “slow” strains (e.g., Alpha) of SARS-CoV-2, immunocompromised individuals (such as pregnant women) are the most active sources of infection;
4. Results of the simulation confirmed that more contagious “fast” strains of the SARS-CoV-2 virus (e.g., Omicron) spread more rapidly in public transportation. For less contagious “slow” strains (e.g., Alpha) of the virus, the highest infection rates occur through work and educational contacts. Therefore, all of these findings underscore the adequacy and accuracy of the model. This implies that the developed model can be further utilized for analyzing and constructing optimal strategies to prevent epidemic spread. It can also be expanded to encompass regions within a country and even worldwide.

## Figures and Tables

**Table 1:** The number of agents working in different job positions.

Object	Workers
Grocery	1000
Offices	3240
Preschools	570
Schools	1330
Retail	500
Transport	100

**Table 2:** Distribution of profession between age groups.

Age Group	Job	Number
Adult	Offices	3141
Elderly	Domestic	1739
Pupil	School Pupil	1109
Adult	Grocery	960
Student	University Student	757
Adult	Retail	487
Adult	Unemployed	328
Adult	Domestic	321
Adult	Schools	271
Children	Preschool Child	244
Children	Domestic	169
Adult	Preschools	116
Adult	Transport	98
Student	Offices	99
Adult	Universities	76
Student	Grocery	40
Student	Retail	13
Student	Unemployed	11
Student	Schools	9
Student	Universities	4
Student	Preschools	4
Student	Transport	2

**Table 3:** The number of days to reach the specified infection level according to the simulation results for the Alpha variant depends on immunity.

	Immunocompetent	Immunocompromised	Difference
25%	32	28	4
50%	37	32	5
75%	44	37	7
86%		43	

**Table 4:** The number of days to reach the specified level of infection for the Delta variant depends on immunity according to the simulation results.

	Immunocompetent	Immunocompromised	Difference
25%	14	12	2
50%	16	14	2
75%	19	16	3
86%		18	

**Table 5:** The number of days to reach the specified level of infection for the Omicron strain depends on immunity according to the simulation results.

	Immunocompetent	Immunocompromised	Difference
25%	6	5	1
50%	7	6	1
75%		9	

## Data Availability

Data are contained within the article.
